# On Coalescence Analysis Using Genealogy Rooted Trees

**DOI:** 10.1155/2014/194202

**Published:** 2014-02-23

**Authors:** Ao Yuan, Gengsheng Qin, Wenqing He, Qizhai Li

**Affiliations:** ^1^Department of Biostatistics, Bioinformatics and Biomathematics, Georgetown University, Washington, DC 20057, USA; ^2^Department of Mathematics and Statistics, Georgia State University, Atlanta, GA 30303, USA; ^3^Department of Statistics and Actuarial Science, University of Western Ontario, London, ON, Canada N6A 5B7; ^4^Academy of Mathematics and Systems Science, Chinese Academy of Sciences, Beijing 100190, China

## Abstract

DNA sequence data are now being used to study the ancestral history of human population. The existing methods for such coalescence inference use recursion formula to compute the data probabilities. These methods are useful in practical applications, but computationally complicated. Here we first investigate the asymptotic behavior of such inference; results indicate that, broadly, the estimated coalescent time will be consistent to a finite limit. Then we study a relatively simple computation method for this analysis and illustrate how to use it.

## 1. Introduction 

In the past decades, considerable progress has been made in the field of population genetics. One of the main goals is to infer the coalescence time of the population under study, that is, to infer the time since their most recent common ancestor (MRCA) and its distribution based on the observed data.

In genetics, coalescent theory is a retrospective of population genetics that traces all genes in a sample from a population to a single ancestral copy shared by all the members of the population. The coalescent time of a population is the time of their most recent common ancestor. The inheritance relationship among the genes is typically represented as a gene genealogy, similar to a phylogenetic tree. The goal of coalescent analysis is to infer the coalescent time of a sample of *n* individuals independently sampled from a population of size *N*, based on their observed DNA sequence diversity. Unlike parameter inference for independent and identically distributed (iid) data, for which asymptotic limit can be used conveniently to characterize the estimator when the data size is large, various existing studies indicate that the estimated MRCA, in unit of *N* generations, is unclear as whether it will concentrate as the data sample size increases without bound. In contrast, in the estimation of mutation rate in the same setting, the estimate is consistent and asymptotically normal [1], although at a much slower rate of log⁡^1/2^(*n*), compared to the rate of *n* ^1/2^ for i.i.d. data. Also, different from usual parameters, the MRCA changes with *n*, the number of sequences. This prompts us to the investigate the asymptotic behavior of the estimated coalescent time. We want to know whether such estimator will be asymptotically consistent and in what sense if it does. Conditioning on the total number of segregating sites, we find that such estimators converge or not to some nonnegative finite limits in posterior mean, depending on the behavior of the number of mutations on all the branches of the rooted trees constructed from the observed data. Also, analysis of this problem with this type of data is often computationally extensive and complicated; we study a relatively simple simulation method for this problem. We first study the asymptotic behavior of this method in Section 3, and then describe and illustrate our method for this problem in Section 4.

In coalescence inference, mitochondrial DNA (mtDNA) data plays an important role. Mitochondria is one of the few genes existing outside the cell nucleus, and for mammalian it is only maternally inherited. Human mtDNA is a double-stranded molecule sequence about 16,500 base pairs in length. It is outside the cell nuclear, and it is known that the mutation rate in mtDNA is about 10 times that of the nuclear genes, and that on one section of the mitochondria, its control region, the mutation rate is even one order higher. The simple inheritance pattern and high variability make mtDNA an important source in the study of human evolutionary history. Each site on the DNA strand has one of the four bases A, C, G, or T. As the molecule evolves, mutations occur in the form of base substitutions. The change between purines (A,G) or pyrimidines (C,T) is called transition; that between a purine and pyrimidine is transversion. The former type of substitution is much more common than the latter.

We focus on the control region of the mitochondrial data in Griffiths and Tavaré [2], which is part of the data in Ward et al. [3]. They are from a segment of the control region, with 352 base pairs (sites), out of which 159 are purine sites and 193 are pyrimidine sites. This data contains 63 sequences sampled from a North American Indian tribe, the Nuu-Chah-Nulth, from Vancouver Island. After eliminating sequences with multiple mutations on some single sites, so that the assumption of at most one mutation each site is met, the remaining data has 55 sequences, with 14 distinct sequences (called lineages) in the data. Site at which not all the observed sequences have the same base is a * segregating site*. The whole sequences are long, but only the segregating sites are informative for the analysis; the other sites are ignored. The mentioned data has 18 segregating sites and is presented in Table 1, with the frequency (or multiplicity) of each lineage.

## 2. Brief Review of Background and Related Methods 

**Figure 1:**
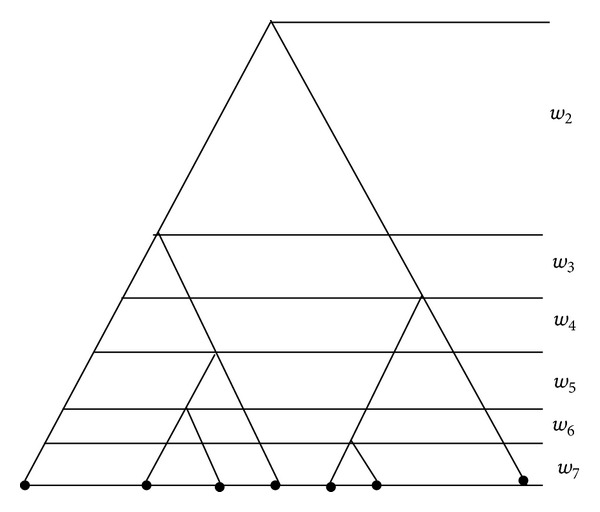
Coalescent tree for a sample of seven individuals.

The coalescent is a model for the genealogical tree of a random sample of *n* DNA sequences from a large population. An example of such a tree of sample size *n* = 7 is given in Figure 1.

For more detailed reviews of this topic, see Hudson [4] and Donnelly and Tavaré [5].

In coalescence inference one has the following.

*Basic Assumptions*. The population size *N* is large, remains unchanged for many generations into the past, and is known, or can be estimated from other sources; the data is a random sample from the population; the number of births in each generation follows the Wright-Fisher model (since the population is of constant size, the number of deaths also follows the similar model); mutation (substitution) at any nucleotide site can occur only once in the ancestry and is irreversible; mutations that occur in different time intervals are independent; the time point at which mutation occurs follows a Poison distribution with rate *θ*/2 to be defined latter, independently in each branch of the genealogy tree, where *θ* is known, or can be estimated from other methods or sources.

The inference of coalescence time *t*_*n*_ of a sample population of size *n* has two steps. The first step is modeling the distribution of *t*_*n*_ without any data, the * predata* distribution; then in the second step, update the predata distribution, using the observed data, to the * postdata* distribution, based on which the formal inference is conducted. The predata distribution is pioneered by Kingman [6, 7]; he showed that, in time units of *N* generations,

(1) $$\begin{array}{c} t_{n}=\overset{n}{\underset{j=2}{\sum }}w_{j}, \end{array}$$

where the *w*_*j*_'s are independent waiting times. *w*_*j*_ is the time from *j* − 1 common ancestors of the sample to *j* common ancestors. A quick reference on this can be found in Tavaré [8]. Here *w*_*j*_ is distributed as exponential Exp(*j*(*j* − 1)/2), with *E*(*w*_*j*_) = 2/(*j*(*j* − 1)). The *w*_*j*_s can be represented graphically as a coalescent tree as in Figure 1; then *t*_*n*_ is the height of the tree. Define the tree length as

(2) $$\begin{array}{c} l_{n}=\overset{n}{\underset{j=2}{\sum }}jw_{j}; \end{array}$$

then (Kingman)

(3) $$\begin{array}{c} E\left(t_{n}\right)=2\left(1-\frac{1}{n}\right),\\ \mathrm{Var}\left(t_{n}\right)=8\overset{n}{\underset{j=2}{\sum }}\frac{1}{j^{2}}-4{\left(1-\frac{1}{n}\right)}^{2};\\ E\left(l_{n}\right)=2\overset{n-1}{\underset{j=1}{\sum }}\frac{1}{j},\\ \mathrm{Var}\left(l_{n}\right)=4\overset{n-1}{\underset{j=1}{\sum }}\frac{1}{j^{2}}. \end{array}$$

The time unit is transformed to years by the relationship *t*_*n*_ *NY*, where *Y* is the average years of each generation, which is usually taken as 20–25. Here we see that, as an initial analysis without the observed data, the coalescent time of a random sample of size *n* from a population of size *N* is roughly 2*N* generations, as long as *n*(≤*N*) is moderately large. Thus, the coalescent time of a sample from a subpopulation is roughly the same as that of the population (as long as the sample size is moderately large). This phenomenon is further investigated by Watterson [9], who showed that

(4) $$\begin{array}{c} P\left(A_{N}\left(t_{n}\right)=1\right)=\frac{\left(n-1\right)\left(N+1\right)}{\left(n+1\right)\left(N-1\right)}, \end{array}$$

where *A*_*N*_(*t*_*n*_) is the number of ancestors, at *t*_*n*_ generations ago, of the population with size *N* from which the data sample of size *n* is drawn. Here the sample must be a random draw from the population; otherwise the result may not be reliable. For example, the sample of size *n* is drawn from a subpopulation of size *N*_1_ < *N* from a population of size *N*; then by (3), the predata estimated of the coalescent time *t*_*n*_ of this sample is roughly 2*N*_1_ generations, but also it is roughly 2*N* generations since the sample is also from the whole population. The paradox arises from the sampling scheme. If the sample is drawn from the subpopulation of size *N*_1_, one can only use 2*N*_1_ as the time scale, not 2*N*, since the samples drawn from the subpopulation are expected to have smaller genetic variation than from the whole population.

For mutation, the common assumption is that the times at which mutation occurs follow a Poison process with constant rate *θ*/2, so that, in any branch of length *l* from the tree, the number of mutations on that branch has a Poison distribution with mean *lθ*/2, independently of the mutations on the other branches. For the time scale mentioned before, usually *θ* = 2*Nμ*, where *μ* is the probability of a mutation that occurs per sequence per generation. For DNA sequences, *μ* is the sequence length (number of bases) times the mutation rate per site per generation and is often available from other sources. Since the coalescent time of a sample with moderate size is approximately 2*N* generations, *θ* can be approximately interpreted as the cumulative (since the time of MRCA) mutation rate (number of mutations) per sequence. Also, since the population size is *N*, *θ*/2 can also be interpreted as the mutation rate of the whole population per generation.

Thus, given the mutation rate *θ* and the tree length *l*_*n*_, the number of mutations *s*_*n*_ in a sample of *n* individuals from the given population follows the Poison distribution Po(*θl*_*n*_/2) [10]

(5) P(sn=k ∣ ln=l)=e−θl/2(θl/2)kk!∶=Po(k,θl2) k=0,1,2,…

Note that this probability does not depend on *n*, but on *k*, *l*, and *θ*. Why *θl*_*n*_/2?. Take *n* = 2; then *θl*_*n*_/2 = *θt*_*n*_ ≈ *θ*, which is the expected number of cumulative mutations since *t*_*n*_ generations ago in a sequence. So *θl*_*n*_/2 is a reasonable choice of the parameter in the Poison distribution. But if we model the number *k* of cumulative mutations per sequence since *t*_*n*_ generations ago, for moderately large *n*, we should use Po(*k*, *t*_*n*_ *Nμ*) ≈ Po(*k*, 2*Nμ*) = Po(*k*, *θ*).

The key in the coalescence inference is to evaluate the postdata distribution of *t*_*n*_, which is much more involved than its predata distribution, it depends heavily on the mutation distribution in the data. For example, if more mutations occur in the earlier stage of the genealogy tree, then the estimated *t*_*n*_ will be bigger. Although under the assumption that mutation can only occur at most once at each site and mutation is irreversible, the total number of mutations in the observed data is just the number of segregating sites. But how the mutations distribute in the branches of the genealogy tree is unknown. Such distribution is crucial in the inference of *t*_*n*_, which depends on how much data information being used and on the actual methods. This is our focus from now on. Denoting by *D*_*n*_ the observed data, the estimated coalescent time ${\hat{t}}_{n}$ of the sample is given by the postdata distribution mean of *t*_*n*_ as

(6) $$\begin{array}{c} {\hat{t}}_{n}=E\left(t_{n}\;|\;D_{n}\right). \end{array}$$

The inference can be viewed as a Bayesian procedure, with the predata and postdata distributions that correspond to the prior and posterior distributions in a Bayesian framework. But unlike the common Bayes setting, here the parameter *t*_*n*_ varies with the sample size *n*, and the data cannot be modeled i.i.d. with this parameter. That is the reason the inference of *t*_*n*_ cannot be made arbitrarily accurate, in the sense that the variance of the postdata distribution cannot be arbitrarily small, as the sample size increases without bound. Also, generally the postdata distribution is not in closed form and has to be evaluated by sampling methods. Tavaré et al. [10] derived the postdata distribution based on only the number of segregating sites in the sample. This method is very convenient to use, but does not use the DNA sequences structural information. The well known method in Griffiths and Tavaré [2], hereafter GT, is based on the full data information represented by a set of rooted trees. This method is one of the basic tools in coalescent inference using full data information, but is computationally complicated.

To evaluate the postdata coalescent distribution, GT used the probabilities recursion formula, derived in Ethier and Griffiths [11]. The method is not easy to fully understand and correctly use for many geneticists. Also these probabilities are computationally prohibitive; the postdata distribution of *t*_*n*_ is computed by a Markov chain Monte Carlo sampling and is quite involved.

Here we study a relatively simple approximate method using the full data information; in this method, instead of computing the tree probabilities as in GT, we just set the post-data tree probabilities as uniform for the *s* + 1 rooted trees and use a simulation method to compute the coalescent distribution; thus, getting round of the complicated evaluations of the tree probabilities, it is easy to understand and much simpler in computation.

The rooted tree plays an important role in the analysis, which is not uniquely determined from the data. The data is equivalent to an unrooted tree, which is equivalent to a set of unrooted trees. Each rooted tree has a 0-1 valued matrix representation which is convenient for some computations, but not any 0-1 valued matrix corresponds to a rooted tree. In the following, we give more details about them and their relationships.

*Rooted Tree*. A rooted tree consists of a system of branches, subbranches, and so forth. The tip of each branch or subbranch represents a known lineage. The observed mutations in the sample are represented as dots in the branches, subbranches, and so forth at specified positions. The observed multiplicity of each lineage is represented as leaves at the tip of each branch or subbranch, and so forth.

The presentation of a rooted tree is unique up to the relative positions of its branches, subbranches, and so forth. A rooted tree has several levels of randomness. If we only know the sample size *n*, then the rooted tree has a total of *n* leaves; apart from that, the shape of the tree, how to split, how to allocate the leaves, how many mutations, and the distribution of the mutations are all random. If the data and the number of mutations are given, then the tree can only take a few shapes. Different from GT and other related literatures, here we put the observed lineage frequencies (multiplicities) as leaves in the corresponding tips of branches, subbranches, and so forth of the rooted tree.

Different from a coalescent tree which has a complete time ordering of the splitting points of branches, a rooted tree has only partial time orderings of these splits and mutations. We only know that splits of branch(es) occurred before those of its subbranches, but do not know the ordering of splits of different branches. We know that mutation(s) on the branch occurred before those on its subbranch(es), but do not know the order of ones on the same branch, same subbranch(es), or on different subbranches. For a given sequence data, it may correspond to more than one different rooted tree. For the observed data in Table 1, all the columns are for segregating sites, and there is no transversion. Under the assumption that mutation can only occur at most once at each site and mutation is irreversible, at each segregating site, one and only one of the base types is mutant; the other type is ancestral. So if we know the mutation status at each segregating site, the mutation statuses are said to be labelled, and we can use a 0-1 valued matrix **X** = (*x*_*ij*_) to denote the observed data, where *x*_*ij*_ = 1, if the base type of lineage *i* at site *j* is mutant, and *x*_*ij*_ = 0 otherwise. Such 0-1 matrix representation of the data is convenient in the analysis. It is easy to see that each rooted tree uniquely determines a 0-1 valued matrix **X**, but an arbitrary 0-1 valued matrix may not correspond to a rooted tree. It must satisfy some conditions to corresponds a rooted tree. There are abundant methods and algorithms on how to judge if a given 0-1 values matrix is a valid representation of a rooted tree, and if so how to build the rooted tree (e.g., [12–16]). We find the method that appeared in a number of articles and is stated as Lemma 1 in Gusfiled [16] is easy to use. Given a valid 0-1 valued matrix **X** (means it satisfies the condition for representing a tree), one can uniquely draw a rooted tree corresponding to it. Here, uniqueness means the genealogy relationships, including which lineages are in the same branch or subbranch, and so forth and which mutation sites are on which section of which branch or subbranch and so forth, are determined, but the particular shape of the tree, such as some branch put on the left or right side, the angle of branches, their lengths, and so forth, are irrelevant. Thus, there is a 1-1 correspondence between a rooted tree and a valid 0-1 valued matrix. Given the observed data, the mutation statuses at the sites are usually unknown. For data with *s* segregating sites, there are 2^*s*^ different ways to labelling the mutation statues, but most of the labeling matrices do not qualify to be representations of a rooted tree; it is known that there are only *s* + 1 different rooted trees, and hence *s* + 1 different labellings (matrices) correspond to the data, and there are existing algorithms to construct the rooted trees and their corresponding matrices (e.g., [16, 17]). However, we find that the method in GT is convenient. By this method, one first needs to construct one rooted tree from the data or its valid 0-1 valued matrix. For example, start from the least shared mutations labeling that, on each column (site) of the data, label the less common base type as mutant (the other as ancestral). It is easy to check the conditions for its validity using Lemma 1 mentioned above. Construct the rooted tree corresponding to this matrix and convert it to an unrooted tree as in GT; that is, absorb those subbranches without mutations into their branch(es), and then straighten the branches, subbranches, and so forth. The unrooted tree is uniquely determined from any of the *s* + 1 rooted trees.

Then, based on this unrooted tree, one can get all the other rooted trees as in Griffiths and Tavaré [18]; that is, alternatively put the tree root point near each of the vertexes that stretch out that vertex, then arrange the branches, subbranches, and so forth into the desired shapes; if there are more than one mutation between two adjacent vertexes, put the tree root point in the middle of two such adjacent mutations, alternatively for all such pairs of mutations, and shape the tree as above. This way we get all the rooted trees from the unrooted tree. In fact, given any rooted tree, all the other *s* rooted trees can be constructed in the same way above, without using the unrooted tree. Once the rooted trees are constructed, the corresponding matrix representations are at hand.

## 3. Asymptotic Behavior of MRCA Estimate

For parameter inference with independent and identically distributed data and sample size *n*, it is known that the estimator is asymptotically consistent and asymptotically normal with rate $\sqrt{n}$. But for inference of MRCA, the data *D*_*n*_ are not independent and identically distributed, and existing studies indicated that the distribution of the estimated MRCA *t*_*n*_ | *D*_*n*_ will not concentrate, even if *n* → *∞*. In the case of estimating the mutation rate with the same data, the estimator is found to be consistent and asymptotically normal with rate log⁡^1/2^(*n*) [1]. This motivates us to investigate the asymptotic behavior of ${\bar{t}}_{n}=E\left(t_{n}|D_{n}\right)$ as a commonly used point estimator of the coalescent time. We want to know whether this estimator has similar asymptotic behavior as the mutation rate estimator. We find that such estimators are not consistent almost surely. To describe the result, we consider the data set in three different commonly used forms. The first type of data we consider is in the form of a coalescent tree as in Figure 1. This type of data is often not practical, as for most real data we do not have the information to construct such tree. But as a starting point it will provide us some guide on the result. There are *n* − 1 nodes (splitting points) in the tree numbered 2 to *n* in their time order. Recall the definition of the *i*th coalescent time *w*_*i*_. Between the (*i* − 1)th and *i*th node there are exactly *i* segments, denote them as *w*_*i*1_,…, *w*_*ii*_ from left to right, each has length *w*_*i*_. Assume the number of mutations *k*_*ij*_ on segment *w*_*ij*_ is known. Let **w** = {*w*_*ij*_ : *i* = 2,…, *n*; *j* = 1,…, *i*.}, **k** = {*k*_*ij*_ : *i* = 2,…, *n*; *j* = 1,…, *i*.} be the mutation distribution corresponding to **w** and *k*_*i*_ = ∑_*j*=1_^*i*^ *k*_*ij*_. Here this type of data is fully represented by **k**. When we do not have **w**, **k** is not uniquely determined. But given each rooted tree **T**_*r*_, **w** and the location information of the mutations, a mutation vector *k*_*r*_ = {*k*_*r*,*i*_ : *i* = 2,…, *n*; *k*_*r*,*i*_ = ∑_*j*=1_^*i*^ *k*_*r*,*ij*_} can be constructed by a random manner (to be detailed in Section 4) corresponding to **T**_*r*_. Denote *π*(**T**_*r*_) = *π*(**T**_*r*_ | *D*_*n*_) = 1/(*s* + 1)  (*r* = 1,…, *s* + 1) be our prior on the rooted tree **T**_*r*_'s, that is, without additional knowledge we treat each rooted tree as equally likely from the observed data. Here our *π*(**T**_*r*_)'s have different meaning from the probabilities *p* ^0^(**T**_*r*_, **n**)'s as in GT (the latter do not sum up to one, but to the probability of obtaining the unrooted tree from the observed data). We have (Appendix)

(7) E(tn ∣ Dn,θ)=∑r=1s+1E[E(tn ∣ kr,Dn,θ)]π(Tr ∣ Dn)=1s+1∑r=1s+1 ∑i=2nE[kr,i]+1i(i+θ−1).

The commonly available data is in the form of Table 1, which is equivalent to *s* + 1 rooted trees; here *s* = |**k** | : = ∑_*i*,*j*_ *k*_*ij*_ = |**k**_*r*_|(*r* = 1,…, *s* + 1).

The last method is to estimate *t*_*n*_ only by the number of mutations *s*, without using the information in the rooted trees.

We have the following result (proof in Appendix).

**Proposition 1**

(i) One has

(8) $$\begin{array}{c} E\left(t_{n}\;|\;k,s,\theta \right)=2\overset{n}{\underset{i=2}{\sum }}\frac{k_{i}+1}{i\left(i+\theta -1\right)}; \end{array}$$

consequently, the above estimator will diverge almost surely, if **k** is treated as random.

(ii) One has

(9) $$\begin{array}{c} E\left(t_{n}\;|\;D_{n},s,\theta \right)=\frac{2}{s+1}\overset{s+1}{\underset{r=1}{\sum }}\;\overset{n}{\underset{i=2}{\sum }}\frac{E\left[k_{r,i}\right]+1}{i\left(i+\theta -1\right)}, \end{array}$$

and ${\hat{t}}_{n}$ will converge or not depending on that of the series above.

(iii) One has

(10) $$\begin{array}{c} E\left(t_{n}\;|\;s,\theta \right)=\frac{2}{s+1}\underset{|k|=s}{\sum }\;\overset{n}{\underset{i=2}{\sum }}\frac{E\left[k_{i}\right]+1}{i\left(i+\theta -1\right)}, \end{array}$$

and the asymptotic behavior of the above estimator depends on the series above.

**Remark 2**

The above result tells us that ${\hat{t}}_{n}$ cannot be characterized by an asymptotic deterministic quantity, even for large data size. The estimator is dominated by the number of mutations in the first few coalescent times. Hence, the only practical way to infer the coalescent time is via numerical methods, as the postdata coalescent distribution has no closed form even asymptotically. In contrast, the predata mean *E*(*t*_*n*_) = 2(1 − 1/*n*) → 2 is convergent but is inaccurate as an estimator of the coalescence time for the population under study.

## 4. The Proposed Method

The method is to construct the mutation vector *k*_*r*_ = (*k*_*r*,2_,…, *k*_*r*,*n*_) and compute the data probability directly from the genealogy rooted tree **T**_*r*_'s. Suppose that there are *s* segregating sites in the sequence data, which is exactly the total number of mutations occurred in the history of the *n* sampled individuals, then there are *s* + 1 different rooted trees **T**_*r*_'s compatible with the data. Each of the rooted trees is a fixed genealogy structure, with the multiplicities as the leaves, but the number of mutations among the tree segments is random, subject to the total number of mutations being *s*. The structure consists of the tree branches, subbranches within each branches, sub-subbranches, and so on,, and the leaves. These are the fixed features of a rooted tree. Given the data, the rooted tree is a display of how the *s* mutations are distributed along the lineages, but there is no time scale in the tree, so (5) cannot be used to compute the mutation probabilities. Each rooted tree tells us a partial ordering of the mutations. For example, in the rooted tree, we know mutations at sites 4, 6, and 14 occurred before the split of lineages *a*, *b*, *e*, and *f*, thus occurred before the mutations at sites 1, 5, and 10. But we do not know which of 4, 6, and 14 occurred first. We know mutation 1 occurred before 10, but we do not know the order of 1 and 5, and so forth. If we have the full data (**k**_*r*_, **w**) corresponding to all the rooted trees, **T**_*r*_'s, we can compute ${\hat{t}}_{n}=E\left(t_{n}|D_{n},\theta \right)$ as in Proposition 1(ii). But **w** and the **k**_*r*_s are not directly available; however, **w** can be easily simulated by the prior exponential distribution, and each rooted tree **T**_*r*_ has an initial mutation distribution on its branch segments. Denote by *s*_*ij*…_ the (*i*, *j*,…)th segment (the order is arbitrary, e.g., we can lable them from upper to lower and left to right locations), and let |*s*_*ij*…_| be the number of mutations on it (many of them are zeros; we can concentrate on the segments with nonzero mutations). Denote **s** = {*s*_*ij*…_}. Given (**w**, **s**), *k*_*r*_ can be sampled from **T**_*r*_ (to be detailed latter). Let *E*_(**w**,**k**_*r*_)_ be the expectation with respect to (**w**, **k**_*r*_). The above motivates us to estimate *t*_*n*_ by

(11) $$\begin{array}{c} {\hat{t}}_{n}=E\left(t_{n}\;|\;D_{n},\theta \right)=\frac{2}{s+1}\overset{s+1}{\underset{r=1}{\sum }}\;\overset{n}{\underset{i=2}{\sum }}E_{\left(w,k_{r}\right)}\left[\frac{k_{r,i}+1}{i\left(i+\theta -1\right)}\right]. \end{array}$$

The above expectation is not easy to compute directly since we do not know the joint distribution of (**w**, **k**_*r*_). Instead we use simulation method. For this, we sample **w** ^(1)^ ⋯ **w** ^(*M*)^ independently and generate *k*_*r*_^(*m*)^ = {*k*_*r*,*i*_^(*m*)^} (see below) corresponding to **w** ^(*m*)^ and **T**_*r*_ for each *r* then approximate ${\hat{t}}_{n}$ as

(12) $$\begin{array}{c} {\hat{t}}_{n}\approx \frac{2}{M}\overset{M}{\underset{m=1}{\sum }}\frac{1}{s+1}\overset{s+1}{\underset{r=1}{\sum }}\;\overset{n}{\underset{i=2}{\sum }}\frac{k_{r,i}^{\left(m\right)}+1}{i\left(i+\theta -1\right)}. \end{array}$$

Now we consider generating *k*_*r*_^(*m*)^. After *w* ^(*m*)^ = (*w*_2_^(*m*)^,…, *w*_*n*_^(*m*)^) is allocated among the branches of **T**_*r*_, we only need to consider each segment *s*_*ij*…_ with nonzero number *t*_*r*_ of mutations in them. Each length of *s*_*ij*…_0 in **T**_*r*_ is the summation of some *d* = *d*_*ij*…_ of the *w*_*j*_^(*m*)^'s. For simplicity of exposition and notation, suppose that they are *w*_2_^(*m*)^,…, *w*_*d*+1_^(*m*)^; then given the *t*_*r*_ mutations in [0, *w*_2_^(*m*)^ + ⋯+*w*_*d*+1_^(*m*)^] and using (5), it is easy to see that the number of mutations ${\tilde{k}}_{r}=\left({\tilde{k}}_{r,1},\ldots ,{\tilde{k}}_{r,d}\right)$ in each of the *d* intervals [0, *w*_2_^(*m*)^], [*w*_2_^(*m*)^, *w*_2_^(*m*)^ + *w*_3_^(*m*)^],…, [*w*_2_^(*m*)^ + ⋯+*w*_*d*−1_^(*m*)^, *w*_2_^(*m*)^ + ⋯+*w*_*d*_^(*m*)^] follows the multinomial distribution

(13) P(k~r=(kr,1,…,kr,d) ∣ tr)  =M(kr,1,…,kr,d;tr,q1,…,qd)  =tr!kr,1!⋯kr,d!q1k1⋯qdkd,

where *q*_*j*_ = *w*_*j*+1_/(*w*_2_ + ⋯+*w*_*d*+1_)  (*j* = 1,…, *d*). After all the nonzero *t*_*r*_ = |*s*_*ij*…_|'s are allocated in the corresponding intervals, we have

(14) $$\begin{array}{c} k_{r,i}^{\left(m\right)}=\underset{\left(i\right)}{\sum }{\tilde{k}}_{r,l},\;\left(i=2,\ldots ,n\right), \end{array}$$

where the summation is for all ${\tilde{k}}_{r,l}$'s that fall in [*w*_2_^(*m*)^ + ⋯+*w*_*i*_^(*m*)^, *w*_2_^(*m*)^ + ⋯+*w*_*i*+1_^(*m*)^].

Specifically, the simulation method is as below. For *m* = 1,…, *M*, do the following steps.

- Sample *w* ^(*m*)^ = (*w*_2_^(*m*)^,…, *w*_*n*_^(*m*)^) from the coalescent distribution as in (1); that is, the *w*_*i*_^(*m*)^'s are independent, with *w*_*i*_^(*m*)^ ~ exp⁡(*i*(*i* − 1)/2). Or equivalently, sample *u* ~ *U*(0,1) and set *w*_*i*_^(*m*)^ = −2/(*i*(*i* − 1))ln⁡(1 − *u*).
- For each fixed 1 ≤ *r* ≤ *s* + 1, allocate *w* ^(*m*)^ to the *n* − 1 coalescent events of the *n* sequences based on each rooted tree **T**_*r*_. See illustration below for details.
- Allocate the ${\tilde{k}}_{r}^{\left(m\right)}$ mutations in the corresponding segments according to (13). Then get the *k*_*r*,*i*_^(*m*)^'s as in (14).

After all the *M* iterations, evaluate (12) until convergence, which can be assessed by relative error, for example.

*Illustration: Allocate* **w** ^(*m*)^ *to the n* − 1* coalescent events of the n sequences based on rooted tree*. We use the backward method; that is, first allocate *w*_*n*_^(*m*)^, then *w*_*n*−1_^(*m*)^,…, and last *w*_2_^(*m*)^. Consider the rooted tree, for example. There are *n* = 55 sequences, with frequencies (3,1, 19,2, 2,1, 5,1, 1,1, 4,8, 8,3) for lineages (*m*, *n*, *e*, *b*, *a*, *f*, *k*, *c*, *h*, *g*, *i*, *j*, *l*, *d*). Note that sequences (leaves) in each lineage (branch) only coalescence within each branch (if the branch has more than one leaves), and branch with a single leaf coalescences only at MRCA *w*_2_^(*m*)^. We first decide *w*_55_^(*m*)^ goes to which branch or pairs of single branches. Since it is the latest coalescent time, it can only go to a pair of leaves in some branch with multiple leaves. Since branch *n* has only 1 leaf; it is excluded at this step. The remaining branches (*m*, 〈*e*, *b*, *a*, *f*〉, *k*, 〈*c*, *h*, *g*, *i*, *j*, *l*〉, *d*) all have multiple leaves with a total of 54. We assign *w*_55_^(*m*)^ to one of these branches with weights proportional to their number of leaves, that is, with probabilities (3,24,5, 19,3)/54. Suppose that *w*_55_^(*m*)^ is assigned to 〈*e*, *b*, *a*, *f*〉; we need to decide which subbranch it goes to. We have three candidate subbranches (*e*, *b*, *a*) with number of leaves (19,2, 2). We randomly assign *w*_55_^(*m*)^ to them with weights (19,2, 2)/23. Suppose it is assigned to branch *e*; then *w*_55_^(*m*)^ will go to a pair within this branch, and which pair is irrelevant. But the pair will be treated as a single leaf in assigning the rest *w*_*j*_^(*m*)^'s. So after this step, we reassign the number of leaves in *e* as 18.

Now we assign *w*_54_^(*m*)^. The procedure is the same as above; the only difference is now *e* has 18 leaves. The candidate branches are still (*m*, 〈*e*, *b*, *a*, *f*〉, *k*, 〈*c*, *h*, *g*, *i*, *j*, *l*〉, *d*) with weights (3,23,5, 19,3)/53. Suppose that *w*_54_^(*m*)^ is also allocated to *e* of branch 〈*e*, *b*, *a*, *f*〉; then *e* has 17 leaves now.

We now allocate *w*_53_^(*m*)^ to candidates (*m*, 〈*e*, *b*, *a*, *f*〉, *k*, 〈*c*, *h*, *g*, *i*, *j*, *l*〉, *d*) with weights (3,22,5, 19,3)/52. Suppose that *w*_53_^(*m*)^ is allocated to 〈*c*, *h*, *g*, *i*, *j*, *l*〉; we need to decide which of the 4 subbranches it will go to. *c* has only 1 leaf and is excluded. So we allocate subbranches (〈*h*, *g*〉, 〈*i*, *j*〉, *l*) with weights (2,12,4)/18. Supposing it goes to 〈*h*, *g*〉, since it has only one pair of leaves, then *h* and *g* are merged as one leaf after this assignment.

Continue this way, until *w*_2_^(*m*)^ is allocated. Then all the branches in this rooted tree have lengths as the *w*_*i*_^(*m*)^'s allocated to them. After this step, the length of each segment *s*_*ij*…_ of **T**_*r*_ is a summation of some *w*_*i*_^(*m*)^'s. Since |*s*_*ij*…_| is known from each **T**_*r*_, we can allocate each of the ${\tilde{k}}^{\left(m\right)}$'s by (13), then get the *k*_*i*_^(*m*)^'s by the formula that follows it. Then compute (12).

The assumption that the population size *N* is constant can be relaxed the same way as in GS and Tavaré et al. [10].

## Figures and Tables

**Table 1:** Nucleotide position in control region.

Site	1	2	3	4	5	6	7	8	9	10	11	12	13	14	15	16	17	18	Lineage
	Purines					Pyrimidines													Freqs.
Lineage																			
a	A	G	G	A	A	T	C	C	T	C	T	T	C	T	C	T	T	C	2
b	A	G	G	A	A	T	C	C	T	T	T	T	C	T	C	T	T	C	2
c	G	A	G	G	A	C	C	C	T	C	T	T	C	C	C	T	T	T	1
d	G	G	A	G	A	C	C	C	C	C	T	T	C	C	C	T	T	C	3
e	G	G	G	A	A	T	C	C	T	C	T	T	C	T	C	T	T	C	19
f	G	G	G	A	G	T	C	C	T	C	T	T	C	T	C	T	T	C	1
g	G	G	G	G	A	C	C	C	T	C	C	C	C	C	C	T	T	T	1
h	G	G	G	G	A	C	C	C	T	C	C	C	T	C	C	T	T	T	1
i	G	G	G	G	A	C	C	C	T	C	T	T	C	C	C	C	C	T	4
j	G	G	G	G	A	C	C	C	T	C	T	T	C	C	C	C	T	T	8
k	G	G	G	G	A	C	C	C	T	C	T	T	C	C	C	T	T	C	5
l	G	G	G	G	A	C	C	C	T	C	T	T	C	C	C	T	T	T	4
m	G	G	G	G	A	C	C	T	T	C	T	T	C	C	C	T	T	C	3
n	G	G	G	G	A	C	T	C	T	C	T	T	C	C	T	T	T	C	1

Each row of the table represents a DNA sequence lineage. In this data, there are transitions but no transversion observed.

## Appendix

### Proof of the Proposition

(i) Recall that the *k*_*ij*_'s are independent, the *k*_*i*_'s are independent, and the *w*_*i*_'s are independent with *w*_*i*_ ~ exp⁡(*i*(*i* − 1)/2), *E*(*w*_*i*_) = 2/(*i*(*i* − 1)/2), *k*_*ij*_ | *w*_*i*_ ~ Po(·, *w*_*i*_ *θ*/2), *k*_*i*_ | *w*_*i*_ ~ Po(·, *iw*_*i*_ *θ*/2), and so *E*(*k*_*i*_) = *E*(*E*(*k*_*i*_ | *w*_*i*_)) = *θ*/(*i* − 1). Observe

(A.1) P(wi ∣ Dn,θ)=P(wi ∣ k,θ)=P(wi ∣ ki,θ)∝P(wi)P(ki ∣ wi,θ)=i(i−1)2exp⁡(−i(i−1)wi2)×Po(ki,iwiθ2)∝(wiθ)kiexp⁡(−i(i+θ−1)wi2).

The right-hand side above is the density of Γ(*k*_*i*_ + 1,2/(*i*(*i* + *θ* − 1)) distribution, up to an normalizing constant. It has mean *E*(*w*_*i*_ | **k**, *θ*) = 2(*k*_*i*_ + 1)/(*i*(*i* + *θ* − 1)) and variance Var⁡(*w*_*i*_ | **k**, *θ*) = 4(*k*_*i*_ + 1)/(*i* ^2^(*i*+*θ*−1)^2^). Since *t*_*n*_ = ∑_*i*=2_^*n*^ *w*_*i*_, we have

(A.2) E(tn ∣ k,θ)=∑i=1nE(wi ∣ k,θ)=2∑i=2nki+1i(i+θ−1)=Sn∑i=2nan,ixi+bn,

where *x*_*i*_ = (*i* − 1)*k*_*i*_, the *x*_*i*_'s are i.i.d with *E*(*x*_*i*_) = *θ*, *a*_*n*,*i*_ = *a*_*n*,*i*_(*θ*) = *S*_*n*_^−1^(*θ*)2/[*i*(*i* − 1)(*i* + *θ* − 1)], *S*_*n*_ = *S*_*n*_(*θ*) = 2∑_*i*=2_^*n*^1/[*i*(*i* − 1)(*i* + *θ* − 1)], and *b*_*n*_(*θ*) = 2∑_*i*=2_^*n*^1/[*i*(*i* + *θ* − 1)]. Note that {*S*_*n*_(*θ*)} and {*b*_*n*_(*θ*)} are convergent sequences with

(A.3) $$\begin{array}{c} S_{n}\left(\theta \right)⟶S\left(\theta \right):=\overset{\infty }{\underset{i=2}{\sum }}\frac{2}{i\left(i-1\right)\left(i+\theta -1\right)}<\infty ,\\ b_{n}\left(\theta \right)⟶b\left(\theta \right):=\overset{\infty }{\underset{i=2}{\sum }}\frac{2}{i\left(i+\theta -1\right)}<\infty . \end{array}$$

Note also that ∑_*i*=2_^*n*^ *a*_*n*,*i*_ = 1, that is, {*a*_*n*,*i*_} is a weight sequence for each fixed *n*. Since lim⁡_*n*_ *a*_*n*,*i*_ > 0 for fixed *i*, by the results for weighted sum of i.i.d. random variables (see [19], for a review of such results), a necessary condition for ∑_*i*=2_^*n*^ *a*_*n*,*i*_ *x*_*i*_ to converge (a.s.) is that lim⁡_*n*_ *a*_*n*,*i*_ = 0 for all fixed *i*, or no term will be dominant as *n* → *∞*. Since this condition is not satisfied, the first few terms are dominant and we have

(A.4) ∑i=2nan,ixi  diverges  (a.s.),or    equivalently  E(tn ∣ k,θ)  diverges  (a.s.).

The proofs of part (ii) and (iii) are similar to that of part (i) and omitted.
